# Supplementary material for: EnzML: multi-label prediction of enzyme classes using InterPro signatures
Source: BMC Bioinformatics. 2012 Apr 25;13:61. doi: 10.1186/1471-2105-13-61 (PMC3483700; doi:10.1186/1471-2105-13-61)
Supplement: Addtional file 5 — The Java code to format the data files, evaluate and predict. The file enzml_java_code.tar.gz contains the Java code used to format database data to ARFF and XML formats, to execute cross and train-test (jackknife) evaluations and to record evaluation results to database. More information is included in the readme.txt file and the Javadoc files. The code can be used with a MySQL database. To use a different database software, other JDBC drivers might be required. [file 1471-2105-13-61-S5.gz › java_code/utils/doc/edu/cornell/lassp/houle/RngPack/RandomShuffle.html]

RandomShuffle


---


|  |  |  |
| --- | --- | --- |
| **Overview** \| **Package** \| **Class** \| **Use** \| **Tree** \| **Deprecated** \| **Index** \| **Help** | |  |
| **PREV CLASS**   **NEXT CLASS** | **FRAMES**    **NO FRAMES**     **All Classes** |  |
| SUMMARY: NESTED \| FIELD \| CONSTR \| METHOD | DETAIL: FIELD \| CONSTR \| METHOD |  |


---


## edu.cornell.lassp.houle.RngPack Class RandomShuffle

```
java.lang.Object
  cern.colt.PersistentObject
      edu.cornell.lassp.houle.RngPack.RandomElement
          edu.cornell.lassp.houle.RngPack.RandomShuffle
```

**All Implemented Interfaces:**: java.io.Serializable, java.lang.Cloneable

---

``` public class RandomShuffle extends RandomElement ```

RandomShuffle uses one random number generator to shuffle the numbers
produced by another to obliterate sequential correlations. To initialize a
RandomShuffle, pass it two RandomElements. The first RandomElement is used to
generate a table of random numbers and the second is used to choose one from
the table. An example of usage is,

```
 RandomElement markov=new RandomShuffle(new Ranecu(),new Ranmar(),32)
```

which would generate a deck of 32 numbers from RANECU and use
RANMAR to choose from the deck.
  
**References:**

- F. James; Comp. Phys. Comm. **60** (1990) p
  329-344- D. Knuth; The Art of Computer Programming vol. 2, sec 3.2.2

Source code  is available.

**Version:**
:   1.0

**Author:**
:   Paul Houle  (E-mail: houle@msc.cornell.edu)

**See Also:**: Serialized Form

---

**Constructor Summary**

`RandomShuffle(RandomElement ga,
RandomElement gb,
int ds)`


| **Method Summary** | |
| --- | --- |
| `java.lang.Object` | `clone()`             Returns a copy of the receiver. |
| `double` | `raw()`             The generator. |

**Methods inherited from class edu.cornell.lassp.houle.RngPack.RandomElement**

`choose, choose, gaussian, gaussian, powlaw, raw, raw, uniform`

**Methods inherited from class java.lang.Object**

`equals, getClass, hashCode, notify, notifyAll, toString, wait, wait, wait`

**Constructor Detail**

### RandomShuffle

```
public RandomShuffle(RandomElement ga,
                     RandomElement gb,
                     int ds)
```

**Parameters:**: `ga` - generator to fill shuffle deck: `gb` - geberator to choose from shuffle deck: `ds` - the size of the shuffle deck


**Method Detail**

### clone

```
public java.lang.Object clone()
```

:   Returns a copy of the receiver.

    :   **Overrides:**: `clone` in class `PersistentObject`
    :   **Returns:**: a copy of the receiver.

---


### raw

```
public double raw()
```

:   The generator.

    :   **Specified by:**: `raw` in class `RandomElement`
    :   **Returns:**: a random double in the range (0,1). **See Also:**: `RandomElement.raw()`


---


|  |  |  |
| --- | --- | --- |
| **Overview** \| **Package** \| **Class** \| **Use** \| **Tree** \| **Deprecated** \| **Index** \| **Help** | |  |
| **PREV CLASS**   **NEXT CLASS** | **FRAMES**    **NO FRAMES**     **All Classes** |  |
| SUMMARY: NESTED \| FIELD \| CONSTR \| METHOD | DETAIL: FIELD \| CONSTR \| METHOD |  |


---
